# Uniparental disomy of the entire X chromosome in Turner syndrome patient-specific induced pluripotent stem cells

**DOI:** 10.1038/celldisc.2015.22

**Published:** 2015-08-25

**Authors:** Yumei Luo, Detu Zhu, Rong Du, Yu Gong, Chun Xie, Xiangye Xu, Yong Fan, Bolan Yu, Xiaofang Sun, Yaoyong Chen

**Affiliations:** 1 Key Laboratory for Major Obstetric Diseases of Guangdong Province, Key Laboratory of Reproduction and Genetics of Guangdong Higher Education Institutes, The Third Affiliated Hospital of Guangzhou Medical University, Guangzhou, China

**Keywords:** induced pluripotent stem cell, Turner syndrome, uniparental disomy, chromosome therapy, X chromosome inactivation

## Abstract

The human induced pluripotent stem cell (iPSC) technique promises to provide an unlimited, reliable source of genetically matched pluripotent cells for personalized therapy and disease modeling. Recently, it is observed that cells with ring chromosomes 13 or 17 autonomously correct the defects via compensatory uniparental disomy during cellular reprogramming to iPSCs. This breakthrough finding suggests a potential therapeutic approach to repair large-scale chromosomal aberrations. However, due to the scarceness of ring chromosome samples, the reproducibility of this approach in different individuals is not carefully evaluated yet. Moreover, the underlying mechanism and the applicability to other types of chromosomal aberrations remain unknown. Here we generated iPSCs from four 45,X chorionic villous fibroblast lines and found that only one reprogrammed line acquired 46,XX karyotype via uniparental disomy of the entire X chromosome. The karyotype correction was reproducible in the same cell line by either retroviral or episomal reprogramming. The karyotype-corrected iPSCs were subject to X chromosome inactivation and obtained better colony morphology and higher proliferation rate than other uncorrected ones. Further transcriptomic comparison among the fibroblast lines identified a distinct expression pattern of cell cycle regulators in the uncorrectable ones. These findings demonstrate that the iPSC technique holds the potential to correct X monosomy, but the correction rate is very low, probably due to differential regulation of cell cycle genes between individuals. Our data strongly suggest that more systematic investigations are needed before defining the iPSC technique as a novel means of chromosome therapy.

## Introduction

Previous studies have demonstrated that cells with supernumerary or reduced number of chromosomes or other chromosomal aberrations can be reprogrammed into induced pluripotent stem cells (iPSCs) by introducing four reprogramming factors [1–3]. These iPSCs with aneuploidy syndromes have served as excellent disease models to deepen our understandings of these syndromes [4–7] and to examine novel therapeutic approaches [8, 9]. Recently, it is observed that ring chromosomes 13 or 17 (referred to r(13) and r(17) in the following) in cells are replaced by compensatory uniparental disomy (UPD) during cellular reprogramming and those key genes in the original heterozygous deletions restore biallelic expression dosages in the resulting iPSCs [10]. This unexpected finding suggests an attractive therapeutic approach for large-scale chromosomal aberrations, as the karyotype correction process is ‘autonomous’ without requiring further genetic modifications like other approaches [8, 9, 11]. It is hypothesized that the UPD is induced by accelerated cell cycle and less efficient decatenation checkpoint in pluripotent stem cells [10]; however, the exact mechanism is not clear yet. Variation of correction efficiency between r(13) and r(17) cell lines, and between different r(13) cell lines is also observed [10], but it is difficult to test the reproducibility in more cell lines from different individuals due to the scarceness of samples. Moreover, the application of this approach is quite limited if it only works for ring chromosomes, which composes merely a tiny fraction of all chromosomal aberrations. Thus, it will be valuable to explore the applicability of this approach in other type of chromosomal aberrations.

**Figure 1:**
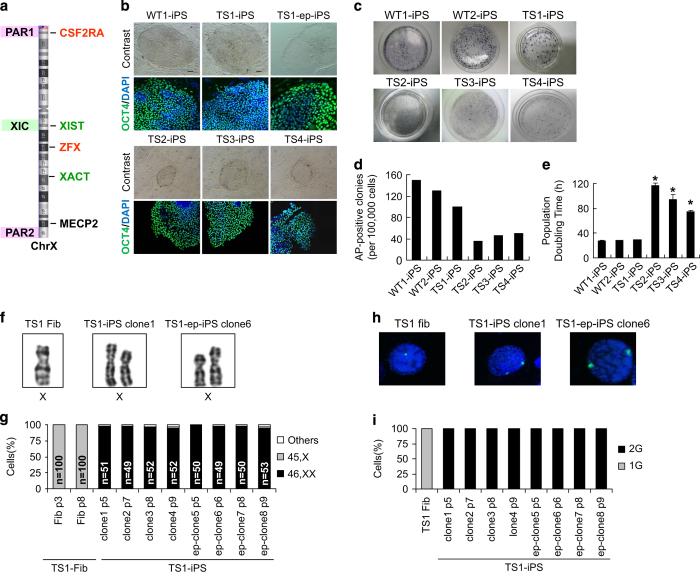
Reprogramming from fibroblasts with 45,X produces iPSCs that acquire normal 46,XX karyotype. (**a**) Schematic diagram of human chromosome X. XIST and XACT are lncRNAs associated with XCI in human pluripotent stem cells. *CSF2RA* and *ZFX* are XCI-escaping genes whose haploinefficiency is associated with TS. *MECP2* is a gene subjected to XCI. (**b**) Colony morphologies and immunostaining of pluripotency marker OCT4 for WT and TS iPSCs. (**c**) AP staining for WT and TS iPSCs. (**d**) Reprogramming efficiency for WT and TS iPSCs. (**e**) Population doubling time for WT and TS iPSCs (*n*=3). Error bars represent s.d. *P<0.001 versus WT1 by *t*-test. (**f**) Representative images of chromosome X pairs in TS1 fibroblasts or iPSC clones. (**g**) Percentage of mitotic cells with one or two chromosome X in TS1 fibroblasts or iPSC clones. (**h**) Signal patterns of FISH probes for chromosome X copy number in TS1 fibroblasts or iPSC clones. (**i**) Percentage of cells with one or two copies of chromosome X signals in TS1 fibroblasts or iPSC clones (*n*=100 each). AP, Alkaline phosphatase; FISH, fluorescence *in situ* hybridization; iPSCs, induced pluripotent stem cells; lncRNAs, long non-coding RNA; PAR, pseudoautosomal region; TS, Turner syndrome; WT, wild type; XIC, X inactivation center.

To probe these issues, we performed factor-based reprogramming on cells with monosomy X, also known as Turner syndrome (TS). TS is the only survivable monosomy syndrome and also one of the most common chromosomal aberrations with an occurrence of up to 3% in all human embryos [12]. Although one of the two X chromosomes in female is inactivated (XCI) to compensate expression dosage and mice with monosomy X is viable and reproducible, >99% of 45,X human embryos die at the early stage and the survival ones suffer from a broad spectrum of symptoms including infertility [13]. It is hypothesized that these symptoms are mostly caused by haploinsufficiency of genes that escape XCI, which compose up to 15% of all X-linked genes and require biallelic expression like autosome genes [14]. Recent studies using human embryonic stem cell (hESC) models has identified haploinsufficiency of the XCI-escaping genes *CSF2RA* and *ZFX* as main reasons for early embryonic lethality and embryo growth defects of TS [15, 16] (Figure 1a). However, owing to the profound and severe effects of whole chromosome loss that simultaneously affects numerous genes, no feasible therapy approach has been proposed yet.

Here we have reprogrammed a total of four TS cell lines with pure 45,X karyotype into iPSCs and applied multiple techniques, including cytogenetics, fluorescence *in situ* hybridization (FISH), DNA fingerprinting, single nucleotide polymorphism (SNP) array so on, to carefully examine their karyotypic alterations as well as the subsequent phenotypic alterations. To gain a preliminary insight into the underlying mechanism, we also profile the global gene expression using transcriptomic arrays. Our data demonstrated that factor-based cellular reprogramming successfully mediated compensatory UPD of the entire X chromosome in one of the TS cell lines, but not for the other three lines, probably owing to the differential expression of cell cycle regulators in these cell lines. It also suggests that the previous ring chromosome study with few cell lines and little investigation on mechanism is insufficient to define factor-based cellular reprogramming as a novel therapeutic strategy for large-scale chromosomal aberrations.

## Results

### Factor-based cellular reprogramming-mediated karyotype correction in TS cells

We generated iPSC lines from two female chorionic villous (CV) fibroblast lines with wild-type 46,XX karyotype (WT1 and WT2) and four lines with pure 45,X karyotype (TS1, TS2, TS3 and TS4) using retrovirus vectors. All TS iPSC lines expressed stem cell markers (Supplementary Figures S1 and S4), hold the potential to differentiate into cell types of the three germ layers (Supplementary Figure S2) and form teratomas *in vivo* (Supplementary Figure S3); however, the colony morphology (Figure 1b), reprogramming efficiency (Figure 1c and d) and proliferation rates (Figure 1e) varied between cell lines. The TS1 iPSC line had the highest reprogramming efficiency among the TS cell lines, and all its clones exhibited proper colony morphology and grew well as the wild-type controls. In contrast, clones from TS2, TS3 and TS4 iPSC lines had flattened morphology and grew much more slowly, with a population doubling time up to 4 days. These clones grew so slowly that we could only passage them twice per month, and several clones even collapsed after three and four passages. Previous studies did not report growth defects in TS iPSCs; however, most of their TS cell lines were derived from survived children, thus representing merely <1% TS patients that can survive to birth. Here our TS cell lines were all derived from prenatal CV fibroblasts; hence, we considered the varied growth phenotypes in our TS iPSC lines reflected the different degrees of embryo growth arrest observed in aborted TS fetuses [17].

We then selected four clones from each cell line for cytogenetic analysis of monosomy X. Surprisingly, we found that all clones from the TS1 iPSC line displayed 46,XX karyotype whereas clones from TS2, TS3 and TS4 iPSC lines maintained the original 45,X karyotype (Figure 1f and g, Supplementary Figure S5). Further analysis of interphase nuclei by FISH confirmed that almost 100% of the TS1 cells had two copies of X chromosome (Figure 1h and i, Supplementary Figure S6). The results showed that only the TS1 cell line acquired normal karyotype after factor-based cellular reprogramming and more interestingly, these karyotype-corrected iPSCs exhibited better phenotypes than other uncorrected ones.

The karyotype alteration of TS1 iPSCs might be explained by either profound effects resulted from random integration of retrovirus vectors into the host genome, or the strong adaptive selection for cells with normal karyotype during the process of reprogramming. To test this, we repeated reprogramming the TS1 fibroblasts with non-integrating episomal vectors (TS1-ep-iPSCs). Consistent with the first reprogramming, all clones derived from the two subsequent batches of TS1 iPSCs presented 46,XX karyotype (Figure 1f–i, Supplementary Figures S5 and S6). As the karyotype correction in TS1 cells was reproducible by either integrating or non-integrating reprogramming method, the random effects of retrovirus integration can be excluded.

### The X disomy in TS1 cells was gained by compensatory UPD

**Figure 2:**
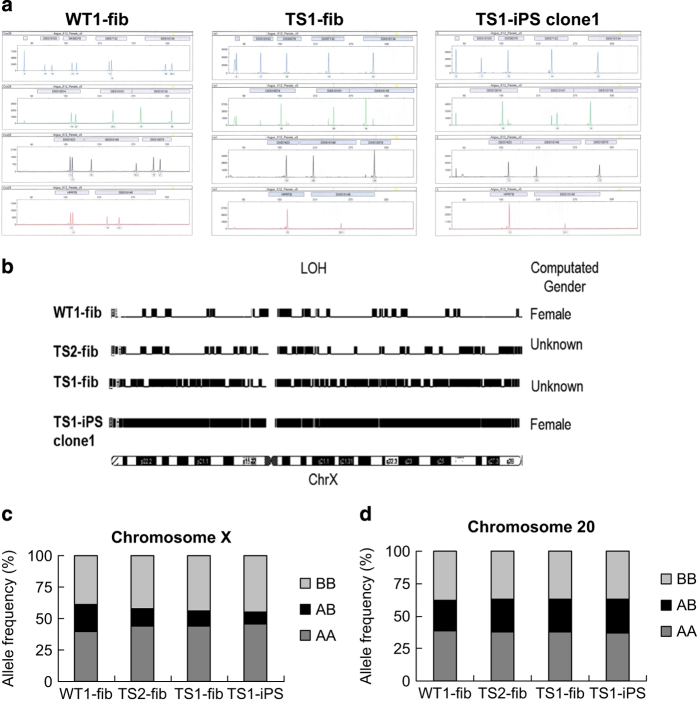
TS1 iPSCs acquired an extra chromosome X via compensatory UPD. (**a**) STR analysis of 13 loci on chromosome X in WT1-fib, TS1-fib and TS1-iPS clone1. (**b**) LOH analysis of chromosome X in WT1-fib, TS1-fib and TS1-iPS clone1. Computated gender: if the mean copy number for the X chromosome is from 0.8 to 1.3 and for Y is from 0.8 to 1.2, then ‘Male’ is assigned; if for X is from 1.9 to 2.1 and for Y is from 0 to 0.4, then ‘Female’ is assigned; if neither of the above cases, then ‘Unknown’ is assigned. (**c** and **d**) Frequency of homozygous or heterozygous SNPs on (**c**) chromosome X or (**d**) chromosome 20 in WT1-fib, TS1-fib and TS1-iPS clone1. iPSCs, induced pluripotent stem cells; LOH, Loss of heterozygosity; SNP, single nucleotide polymorphism; STR, short tandem repeat; TS, Turner syndrome; WT, wild type.

To figure out the source of the X disomy gained in the TS1 iPSC clones, we performed DNA fingerprinting analysis of 13 loci on the X chromosome in WT1-fib, TS1-fib and TS1-iPS clone1. Consistent with compensatory UPD, the two X chromosomes in the TS1 iPSCs are homozygous (Figure 2a, Supplementary Table S3). To avoid the possibility of segmental UPD, SNP microarrays was also performed to WT1-fib, TS2-fib, TS1-fib and TS1-iPS clone1. Principal component analysis of SNPs on the 22 autosomes confirmed that TS1-fib and TS1-iPS clone1 were derived from the same individual (Supplementary Figure S7). Analysis of homozygosity for SNPs on X chromosome showed that the X disomy in the TS1 iPSCs had loss of heterozygosity across the whole chromosome (Figure 2b–d). Taken together, these results demonstrated that the TS1 iPSCs gained X isodisomy by compensatory UPD.

### Phenotype restoration in the TS1 iPSCs

**Figure 3:**
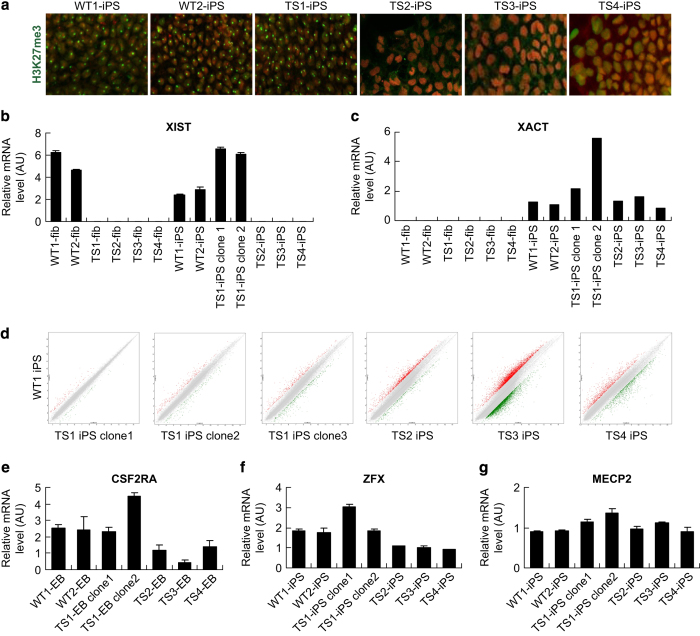
XCI pattern and X-linked gene expression dosages in TS1 iPSCs. (**a**) Immunostaining for Xi marker H3K27me3 in WT and TS iPSCs. (**b** and **c**) qPCR analysis for (**b**) XIST and (**c**) XACT expression in WT and TS fibroblasts or iPSCs. (**d**) Scatter plots comparing genome-wide gene expression patterns between WT and TS iPSCs. (**e**–**g**) qPCR analysis for expression dosages of X-linked genes (**e**) *CSF2RA*, (**f**) *ZFX* and (**g**) *MECP2*. H3K27me3, histone 3 lysine 27 trimethylation; iPSCs, induced pluripotent stem cells; qPCR, quantitative PCR; TS, Turner syndrome; WT, wild type; XIST and XACT are lncRNAs associated with XCI in human pluripotent stem cells; XCI, X-chromosome inactivation.

The acquired X isodisomy in TS1 iPSCs may confer the cells a growth advantage so that they quickly take over the population at early passages. Similarly, it was reported that hESCs frequently gain trisomy X after prolonged culture [18]. Also, X chromosome in female cells undergoes dynamic changes of status throughout reprogramming and prolonged culture [19]. Thus, it is critical to make clear of the XCI status and expression doses of X-linked genes in the TS1 iPSCs. To probe the XCI status, we performed immunostaining of histone 3 lysine 27 trimethylation, a marker for the inactive X chromosome (Xi), and found that there was one Xi signal detected in each cell of the WT1, WT2 and TS1 iPSCs but none in TS2, TS3 and T4 iPSCs (Figure 3a). Then, we further examined the gene expression of XIST, the long non-coding RNA that coats Xi, and XACT, a long non-coding RNA that coats the active X chromosome (Xa) specifically in human pluripotent stem cells [20, 21], by quantitative PCR (qPCR). The results showed that both XIST and XACT were expressed in WT1, WT2 and TS1 iPSCs, but only XACT was expressed in TS2, TS3 and TS4 iPSCs (Figure 3b and c). These data demonstrated that the acquired X isodisomy in TS1 iPSCs underwent XCI, indicating that the X chromosome duplication should happen at an earlier stage and then probably confer the cells a selective advantage.

Besides XCI test, gene expression profiling using transcriptomic arrays was performed to examine the genome-wide effects of the acquired X isodisomy. The results showed that multiple clones from TS1 iPSCs have a more similar expression profile with the WT iPSCs than other uncorrected TS iPSCs (Figure 3d, Supplementary Figure S8). Next, we further examined expression doses of several XCI-escaping and XCI-affected genes that are reported to be important for pluripotent stem cell maintenance or embryo development by qPCR. *CSF2RA* and *ZFX* are XCI-escaping genes that express biallelically; haploinsufficiency of these two genes is probably responsible for the early lethality and developmental defects in 45,X embryos [15, 22]. *MECP2* is a well-known gene subject to XCI and has a role in gene regulation and XCI maintenance in human pluripotent stem cells [23]; however, two copies of active *MECP2* genes resulted from functional X disomy will lead to severe neurodevelopmental abnormalities [24]. Here our qRCP results showed that, consistent with microarray data, the expression doses of CSF2RA at embryoid body stage and ZFX at undifferentiated stage in the TS1 iPSCs raised to a level similar with those of the WT group (WT1 and WT2 iPSCs) and approximately twice higher than those in the uncorrected TS group (TS2, TS3 and TS4 iPSCs) (Figure 3e and f). Meanwhile, the expression dose of MECP2 in the TS1 iPSCs was kept at the same level with those of the WT and uncorrected TS groups (Figure 3g). As ZFX is reported to be essential for hESC self-renewal [16], the results supported our previous observation that the proliferating rate and colony morphology of the TS1 iPSCs were much better than those of the uncorrected TS group.

### The uncorrectable TS fibroblasts had enrichment of cell cycle genes

**Figure 4:**
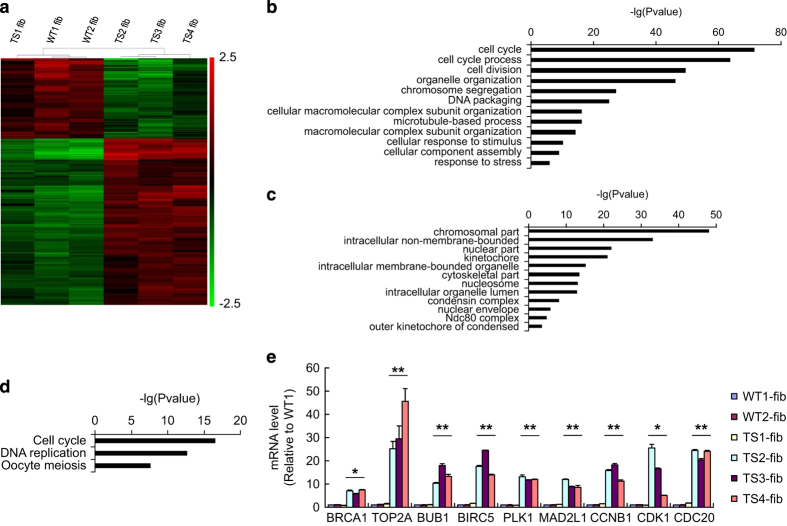
The uncorrectable TS fibroblasts displayed a distinctive cell cycle gene expression pattern. (**a**) Heatmap for differential gene expression profiles between WT group (WT1 and WT2), TS1 and unalterable TS group (TS2, TS3 and TS4) fibroblasts. (**b**) GO biological process and (**c**) cellular component and (**d**) KEGG pathway enrichment analyses for the differentially expressed gene set of the uncorrectable TS group. (**e**) qPCR analysis for differentially expressed cell cycle genes in WT group, TS1 and uncorrectable TS group fibroblasts. Error bars represent s.d. *P<0.05, **P<0.01, uncorrectable TS group versus WT+TS1 group by ANOVA. ANOVA, Analysis of variance; GO, Gene ontology; iPSCs, induced pluripotent stem cells; qPCR, quantitative PCR; TS, Turner syndrome; WT, wild type; XCI, X-chromosome inactivation.

In order to gain a preliminary insight into the underlying mechanism, we performed comparative transcriptomic analysis between WT group (WT1 and WT2 fibroblasts), the karyotype-correctable TS1 fibroblasts and the uncorrectable TS group (TS2, TS3 and TS4 fibroblasts). Clustering analysis showed the uncorrectable TS group had a different expression profile with the WT group and TS1 fibroblasts (Figure 4a). Gene ontology and pathway enrichment analysis found the 312 differentially expressed genes in the uncorrectable TS group were significantly enriched in biological processes like mitotic cell cycle, chromosome segregation and DNA packaging (Figure 4b), in cellular components like kinetochore, chromatin and spindle (Figure 4c), and in pathways like cell cycle, DNA replication and oocyte meiosis (Figure 4d). All these genes were expressed 2–8-folds higher in the uncorrectable TS group than in other groups, including key genes regulating spindle assembly checkpoint, such as BUB1, MAD2L1, AURKA, PLK1, CCNA2, CDK2, CENPE, CDC20 and BIRC5 [25–28], and those regulating the decatenation checkpoint, such as BRCA1, CCNB1, CDK1 and TOP2A [29], indicating a fundamentally different control of cell cycle checkpoints in the cell lines of this group. The gene expression levels were confirmed by qPCR (Figure 4e). We further examined the expression levels of these cell cycle genes in our iPSC lines and found that they were also differentially regulated in the uncorrected TS iPSCs (Supplementary Figure S9). This expression signature may help us to identify and classify CV fibroblasts that have propensity for karyotype correction during factor-based reprogramming.

## Discussion

In this study, we have demonstrated that the karyotype of one 45,X CV fibroblast line, TS1, was autonomously corrected after factor-based cellular reprogramming. Similar with the previous study using ring chromosome cells, the karyotype correction was facilitated via compensatory UPD and those important genes requiring biallelic expression restore normal dosages. Furthermore, we observed that proliferation rate and morphology of the corrected TS1 iPSCs were improved probably due to restoration of the XCI-escaping gene *ZFX*. An additional difficulty of repairing X monosomy is that the X disomy not only need to be replenished, but also need to undergo XCI to compensate the dosages of genes that require monoallelic expression. Our results showed that the X disomy gained in the corrected TS1 iPSCs was subject to XCI and thus would not introduce new problems. Hence, the TS1 iPSCs were successfully repaired at both genomic and epigenomic levels.

However, the potential of using iPSC technique as a therapeutic approach for large-scale chromosomal aberrations remains questionable. A major issue is the variable correction rates between different aberration types. In our study, only one out of four TS fibroblast lines was successfully corrected. Considering such correction event was not reported by other studies on TS iPSCs, the actual correction rate might be even <25%. The correction rate of cells with ring chromosomes seemed pretty good in the previous study [10]; however, great variation of correction efficiency was observed between the one r(17) and two r(13) cell lines used. All r(17)-derived iPSC clones became dominantly or partially euploid at early passages. In contrast, only half of the r(13)-derived iPSC clones became euploid-dominant at early passages. At later passages, one r(13)-derived iPSC line had more euploid-dominant clones than the other line. Hence, it is doubtful whether the correction rate in ring chromosome cells is still 100% if tested in a larger sample size.

The second major issue is the underlying genetic mechanism of reprogramming-mediated UPD. It is still unclear whether reprogramming into pluripotency just provides a selective pressure for pre-existing UPD cells that have a growth advantage, or it also has a role in detecting the genomic imbalance and elevating the occurrence of UPD. In the first scenario, it will require pre-existence of cells with compensatory UPD in the somatic cell line and these cells should have superior growth advantage over the aberrant cells. In our study, pure 45,X cell lines examined by multiple techniques were used; however, it still cannot exclude the possibility of cryptic mosaic (<1%). It is possible that there has extremely low proportion of UPD(X) cells in the TS1 fibroblast line but not in other TS lines; therefore, only the TS1 line was successfully corrected. In the previous study, all ring chromosome fibroblast lines used are mosaic, and not a large number of cells were counted in the cytogenetic and FISH experiments [10]. Hence, it is even more difficult to tell whether there is mosaicism of UPD(13) and UPD(17) cells in the original fibroblast lines, which could be a reason for that all these three cell lines were successfully corrected but with variable correction efficiencies. It will be a great limitation if it requires pre-existing euploid cells for reprogramming-mediated karyotype correction.

In the second scenario, it will require endogenous cell cycle machinery to participate. By comparing the karyotype-correctable and uncorrectable TS fibroblast lines, we have distinguished a panel of cell cycle genes, including key cell cycle checkpoint regulators BRCA1, AURKA, PLK1, BIRC5, CCNA2, CCNB1, CDK1, CDK2 so on, are dysregulated in the uncorrectable cell lines. Interestingly, it was reported that iPSCs frequently gained trisomy 12 during reprogramming and prolonged culture, which resulted in enrichment of cell cycle-related genes [30]. Hence, the elevated expression of cell cycle regulators in the uncorrectable TS group might relax the cells from selective pressure during reprogramming, though more in-depth investigation is needed to draw definite conclusions. Moreover, these differential expressed genes could potentially serve as biomarker to identify patient samples that could go through UPD by cellular reprogramming.

Another concern is that UPD may cause secondary diseases through homozygosity for a recessive mutation or disruption of normal imprinting patterns [31]. There has been a case report of Duchenne muscular dystrophy caused by homozygosity for a deletion of exon 50 of the dystrophin gene in a female with natural UPD(X) [32]. Moreover, two active X chromosomes in female, also known as functional X disomy, will cause unexplained mental retardation. There are also reports of functional X disomy caused by UPD in TS patients [33, 34]. Hence, even if X monosomy can be repaired via reprogramming-mediated UPD, the corrected iPSCs should be carefully examined for X-linked disease mutations and XCI status before use.

In addition, given the parallels between cellular reprogramming process and tumorigenesis [35], this study will also provide an important foundation to reveal the mechanism leading to the high frequency of acquired UPD in cancer cells, which in return contribute greatly to tumor development via homozygosing oncogene mutations and increasing genome instability [36]. Especially, loss of Xi and gain of an extra Xa via UPD is frequently found in breast cancer cells [37, 38] and other female cancers [39]. Therefore, investigation of the causal genetic factors of UPD is also important for developing therapeutic targets for tumor gene therapy [40–43].

## Materials and Methods

### Derivation of patient-specific and WT fibroblasts

After obtaining the informed consent of the patients and the approval of the Ethics Committee of The Third Affiliated Hospital of Guangzhou Medical University, fibroblast lines were derived from prenatal diagnosis CV cells of 12-gestational-week-old using protocols approved by the Institute of Gynecology and Obstetrics, the Third Affiliated Hospital of Guangzhou Medical University (Guangzhou, China). After 1 or 2 weeks, fibroblasts outgrowths from the explants were passaged using trypsin.

### Generation of iPSCs with retroviral vectors

Fibroblasts were infected with viral supernatants generated by transfection of HEK293T cells using Lipofectamine 2000 (Invitrogen, Carlsbad, CA, USA) with retroviral pMXs vector (AddGene, Cambridge, MA, USA) containing the cDNAs of human OCT4, SOX2, KLF4 and c-MYC. Two successive rounds of infection were performed (12 h each); 10 μg ml^−1^ polybrene (Sigma, St Louis, MO, USA) was added to increase infection efficiency. After the second round of infection, the culture medium was exchanged to fresh fibroblast medium (Dulbecco’s modified Eagle’s medium (Gibco, Carlsbad, CA, USA) supplemented with 15% fetal bovine serum (Hyclone, Logan, UT, USA), 1% non-essential amino acids (Gibco), 1 mM L-glutamine (Gibco)). Infection efficiency was monitored separately and was close to 100%, as demonstrated by co-transduction with green fluorescent protein-expressing vectors. On day 3 or 4, the cells were trypsinized, and 5×10^5^ cells were seeded onto mouse embryonic fibroblast feeder layer in a 10-cm culture dish with hESC medium (knockout Dulbecco’s modified Eagle’s medium (Gibco) supplemented with 15% knockout serum replacement (Gibco), 5% fetal bovine serum (Gibco), 0.1 mM non-essential amino acids, 0.1 mM 2-mercaptoethanol (Gibco), 2 mM L-glutamine, 1 000 units ml^−1^ human leukemia inhibitory factor (Chemicon, Temecula, CA, USA) and 10 ng ml^−1^ basic fibroblast growth factor (Invitrogen)). The medium was changed every day. From days 16–20, hESC-like colonies emerged were picked mechanically and expanded in hESC medium on feeders. After 4 and 5 days of culture, colonies were mechanically dispersed into two and three small clumps using a micropipette. The clumps were then transferred to a fresh mouse embryonic fibroblast feeder layer. These cells were again mechanically dissociated during the initial passages. After five passages, they were incubated in 1 mg ml^−1^ collagenase IV (Gibco) for 20–25 min before further culture on freshly prepared feeders. The culture medium was changed every day.

### Generation of human iPSCs with episomal vectors

For non-integrating reprogramming, oriP/EBNA1-based pCEP4 episomal vectors (Invitrogen) expressing Oct4, Sox2, Klf4, L-Myc and Lin28 were cotransfected into 10^6^ cells using Amaxa Nucleofector (Lonza, Basel, Switzerland). The cells were then plated onto a matrigel-coated 10-mm dish and cultured in fibroblast medium. After 24 h, the medium was replaced by N2B27 medium supplemented with 100 ng ml^−1^ basic fibroblast growth factor. The medium was changed every other day, up to 15 days post-transfection. Then the medium was replaced by mTeSR1 (STEMCELL Technologies, Vancouver, Canada) and changed every day. The hESC-like colonies emerged were picked onto new Matrigel-coated dishes for expansion and characterization. Established human iPSC lines were cultured in mTeSR1 medium on dishes coated with Matrigel (BD, Franklin Lakes, NJ, USA).

### Alkaline phosphatase staining and immunostaining

For Alkaline phosphatase staining, cells were fixed with 90% alcohol for 2 min and washed three times with PBS and stained with BCIP/NBT for 30 min in the darkness. For immunostaining, cells were fixed in 4.0% paraformaldehyde for 20 min, permeabilized with 0.5% Tween-20 for 30 min, incubated with primary antibody overnight and incubated with secondary antibody (Invitrogen) for 1 h. The cells were imaged with an inverted confocal microscope The primary antibodies used in this study were SSEA-3 (1:100, Chemicon), SSEA-4 (1:100, Chemicon), TRA1–60 (1:200, Chemicon), TRA1–81 (1:500, Chemicon), OCT4 (1:500, Abcam, Cambridge, MA, USA), alpha-fetoprotein (1:500, Chemicon), nestin (1:100, Millipore, Temecula, CA, USA), alpha-smooth muscle actin (1:500, Chemicon), and histone 3 lysine 27 trimethylation (1:200, Abcam). 4,6-Diamidino-2-phenylindole and propidium iodide (1:500, Sigma) were used for nuclear staining.

### Population doubling time

Cells for population doubling were removed from the plates using dispase, counted and plated onto preseeded 35-mm feeder plates at a density of 100 000 cells per plate onto six plates (two time points in triplicate) for each line in hESC medium. One set was counted after 96 h to give the baseline count and the next set of plates was counted after 144 h to give the intervening growth. Population doubling was calculated following the formula:

$$T_{D}(h)=48\left(\frac{\mathrm{lg}2}{\mathrm{lg}N_{6}-\mathrm{lg}N_{4}}\right)$$

*T*_D_, population doubling time; *N*_6_, cell number at day 6; *N*_4_, cell number at day 4.

### Karyotype analysis

For cytogenetic analysis, iPSCs obtained after every 10 passages were incubated in culture medium with 0.25 g ml^−1^ colcemid (Gibco) for 3 h, harvested, and incubated in 0.4% sodium citrate, 0.4% chloratum: kaliumat (1:1, v/v) at 37 °C for 5 min and then fixed in methanol:acetic acid (3:1, v/v) three times. After Giemsa staining, at least 20 splitting cells were examined in each group for cytogenetic analysis.

### Fluorescence *in situ* hybridization

For FISH analysis, iPSC suspensions were dropped onto wet slides, dried at 63 °C overnight and then dehydrated with ethanol in sequential concentrations of 70, 85 and 100% before hybridization. FISH was performed using Vysis MultiVysion PGT Multi-color Probe Set (Vysis, Downers Grove, IL, USA), which includes five probes for chromosomes of X and Y. The samples were stained according to recommended FISH protocols from manufacturer and examined under a fluorescence microscope. At least 10 cells were examined in each cell line at each time of examination.

### Differentiation of iPSCs *in vitro* and *in vivo*

To investigate the pluripotency of iPSCs *in vitro*, all iPSC colonies were generated from the feeder layers following 1 mg ml^−1^ collagenase IV (Gibco) treatment. The suspension was cultured on bacterial culture plates to allow aggregation and to prevent adherence to the plate. The embryonic bodys culture medium was changed every other day. After suspension culture for 5 days, embryonic bodys formations were examined. The embryonic bodys were transferred to 0.1% gelatin-coated culture dishes for spontaneous differentiation. After 14 days of differentiation, immunostaining was performed using antibodies against alpha-fetoprotein, alpha-smooth muscle actin and nestin.

The TS-iPSCs were dispensed into 300–400 small colonies and injected into the inguinal grooves of 6-week-old severe combined immunodeficiency (two or more mice per cell line). After injection for 4 weeks, the tumors could be found and 8 weeks later, the resultant tumors were removed, fixed for 4–8 h in 4% paraformaldehyde and embedded in paraffin. After staining with hematoxylin and eosin, the sections were examined for the presence of tissues derived from the three germ layers under a light microscope.

### Quantitative PCR

Total RNA was extracted using TRIzol (Invitrogen) according to the manufacturer’s protocol. First-strand cDNA was synthesized using the iScript cDNA Synthesis Kit (Bio-Rad). 1 μl of cDNA reaction mix was subjected to PCR amplification using Applied Biosystems Power SYBR Green PCR Master Mix (Applied Biosystems, Foster, CA, USA) in the Applied Biosystems 7500 Real-Time PCR System (Applied Biosystems). The forward and reverse primers for qPCR analysis were listed in Supplementary Table S1. GAPDH was selected as the internal reference gene for PCR quantification.

### DNA fingerprinting analysis

Total DNA was extracted using the Qiagen DNeasy Tissue Kit (Qiagen, Hilden, Germany). Extracted DNA was amplified for 16 different genetic loci using the Promega Power Plex 16 System kit (Promega, Madison, WI, USA). Capillary electrophoresis was carried out on an automated ABI 3100 Genetic Analyzer (Applied Biosystems). All the established iPSC lines in this study were confirmed to be identical with their original fibroblasts by DNA finger printing (Supplementary Table S2).

### SNP genotyping

Sample DNA was digested by two restriction enzymes NspI (New England Biolabs, Ipswich, MA, USA) and StyI (New England Biolabs) separately and two adaptors were ligated to the DNA fragment to perform a PCR amplification. Amplified DNA was labeled using Affymetrix Genome-Wide Human SNP Nsp/Sty Assay Kit 5.0/6.0 (Affymetrix, Santa Clara, CA, USA) following the manufacturer’s instructions to obtain biotin-labeled DNA. Array hybridization was performed at 50 °C in Hybridization Over (Affymetrix). After 16 h hybridization, arrays were washed in Fluidics Station (Affymetrix). Arrays were scanned by GeneChip Scanner 3000 (Affymetrix) and Command Console Software 3.1 (Affymetrix) with default settings. Raw data passed quality control were further analyzed by Genotyping Console Software (Affymetrix) to obtain genotype call of each SNP locus.

### Transcriptomic microarray analysis

Total RNA were amplified, labeled and purified using Affymetrix WT Amplication Kit (Affymetrix) and GeneChip WT Terminal Labeling Kit (Affymetrix) to obtain biotin-labeled cDNA. Array hybridization and wash was performed using GeneChip Hybridization Wash and Stain Kit (Affymetrix) in Hybridization Oven 645 (Affymetrix) and Fluidics Station 450 (Affymetrix). Slides were scanned by GeneChip Scanner 3000 (Affymetrix) and Command Console Software 3.1 (Affymetrix) with default settings. Raw data were normalized by Gene Spring Software 11.0 (Agilent Technologies, Palo Alto, CA, USA).
