# Supplementary material for: Uniparental disomy of the entire X chromosome in Turner syndrome patient-specific induced pluripotent stem cells
Source: Cell Discov. 2015 Aug 25;1:15022–. doi: 10.1038/celldisc.2015.22 (PMC4860828; doi:10.1038/celldisc.2015.22)
Supplement: Supplementary Tables [file celldisc201522-s1.doc]

**Supplementary Table 1** **qPCR primer sets**

| Gene name | Primer sequence (5' to 3') |
| --- | --- |
| en-hc-MYC | F:CGAGAGGACCCGTGGATGCAGAG |
|  | R:TTGAGGGGCATCGTCGCGGGAGGCTG |
| en-hOCT4 | F:GACAGGGGGAGGGGAGGAGCTAGG |
|  | R:CTTCCCTCCAACCAGTTGCCCCAAAC |
| en-hSOX2 | F:GGGAAATGGGAGGGGTGCAAAAGAGG |
|  | R:CGTGAGTGTGGATGGGATTGGTG |
| en-hKLF 4 | F:ACGATCGTGGCCCCGGAAAAGGACC |
|  | R:TGATTGTAGTGCTTTCTGGCTGGGCTCC |
| en-hNANOG | F:CAGCCCCGATTCTTCCACCAGTCC |
|  | R:CGGAAGATTCCCAGTCGGGTTCACC |
| en-hREX1 | F:CAGATCCTAAACAGCTCGCAGAAT |
|  | R:GCGTACGCAAATTAAAGTCCAGA |
| tg-hOCT4 | F:CCCCAGGGCCCCATTTTGGTACC |
|  | R:TTATCGTCGACCACTGTGCTGCTG |
| tg-hSOX2 | F:GGCACCCCTGGCATGGCTCTTGGCTC |
|  | R:TTATCGTCGACCACTGTGCTGCTG |
| tg-hKLF 4 | F:ACGATCGTGGCCCCGGAAAAGGACC |
|  | R:TTATCGTCGACCACTGTGCTGCTG |
| tg-hc-MYC | F:CAACCGAAAATGCACCAGCCCCAG |
|  | R:TTATCGTCGACCACTGTGCTGCTG |
| XIST | F:CCATTCTTGCCCACCTGT |
|  | R:TGCCAACCTTCCTTCCTG |
| XACT | F:GAGCAAAATCAAAGGCAGGT |
|  | R:GCCAAGCCAGTAATGGGATA |
| CSF2RA | F:CGAAACTCAAAGAGAAGG |
|  | R:GTGTCCAAAAGTGAATCAA |
| ZFX | F:ATGGAAGAAGCAGATGTGTC |
|  | R:TTGAGGCTGAAGTAATGTCA |
| MECP2 | F:CCCAAGGAGCCAGCTAAGACT |
|  | R:GGAGGATGAAACAATGTCTTTGC |
| AURKA | F:CAGTCTTAGGAATCGTGC |
|  | R:GCTTTCTGAATAGGGAGG |
| PLK1 | F: CTACATCGACGAGAAGCGGG |
|  | R:GTCCACCATAGTGCGGGC |
| TOP2A | F:ACATTCCTATTATTCCCAT |
|  | R:AGGTTCTTCTCCATCCA |
| CENPE | F:AAGGAGAGTAGAGTTCAAGGTT |
|  | R:ACTGTGTAGTTGCTAGGTCATC |
| CCNB1 | F:GTTACTGAAGGTGATGGAGGT |
|  | R:GAAACAGAAAACAGGGGGA |
| CDK1 | F:CCGCAACAGGGAAGAACAG |
|  | R:CGAAAGCCAAGATAAGCAACT |
| BRCA1 | F: TAGCAAGGAGCCAACAT |
|  | R:TTTCTCTCACACAGGGG |
| GAPDH | F:TGACTTCAACAGCGACACCCA |
|  | R:CACCCTGTTGCTGTAGCCAAA |

F: forward primer; R:reverse primer

**Supplementary Table 2** **DNA fingerprinting for cell lines used in this study**

| Loci | WT1 | WT2 | TS1 | TS2 | TS3 | TS4 |
| --- | --- | --- | --- | --- | --- | --- |
| D8S1179 | 12/12 | 13/16 | 12/14 | 14/14 | 10/16 | 13/14 |
| D21S11 | 29/32 | 30/31 | 28/31.2 | 30.2/31 | 28/33.2 | 28.2/31.2 |
| D7S820 | 11/11 | 8/8 | 8/13 | 9/11 | 8/9 | 9/10 |
| CSF1PO | 12/12 | 11/11 | 10/11 | 12/12 | 12/12 | 9/12 |
| D3S1358 | 16/17 | 15/16 | 16/17 | 15/18 | 13/17 | 15/16 |
| THO1 | 6/10 | — | 9/9 | — | — | — |
| D13S317 | 8/8 | 9/12 | 8/14 | 10/12 | 8/10 | 8/12 |
| D16S539 | 9/10 | 11/11 | 9/11 | 9/11 | 9/11 | 11/12 |
| D2S1338 | 19/22 | 20/25 | 22/23 | 19/21 | 19/23 | 19/25 |
| D19S433 | 13/13 | 14.2/16.2 | 13/14 | 13/16.2 | 12/14 | 13/15.2 |
| vWA | 17/17 | 14/16 | 14/16 | 14/17 | 14/18 | 18/19 |
| TPOX | 8/9 | — | 8/8 | — | — | — |
| D12S391 | — | 20/20 | — | 17/22 | 18/22 | 21/22 |
| D18S51 | 15/17 | 15/16 | 14/16 | 13/13 | 12/13 | 14/14 |
| D5S818 | 10/11 | 11/11 | 10/12 | 10/11 | 11/12 | 11/12 |
| D6S1043 | — | 18/19 | — | 13/18 | 13/13 | 13/18 |
| FGA | 22/22 | 19/24 | 23/28 | 24/27 | 18/23 | 20/23 |
| Amelogenin | X | X | X | X | X | X |
| Confirmed samples | WT1-fib p6  WT1-iPS clone1 p9 | WT2-fib p6  WT2-iPS clone1 p9 | TS1-fib p3  TS1-iPS clone1 p5  TS1-iPS clone2 p7  TS1-ep-iPS clone5 p5 | TS2-fib p5  TS2-iPS clone1 p8  TS2-iPS clone2 p9 | TS3-fib p5  TS3-iPS clone1 p8  TS3-iPS clone2 p9 | TS4-fib p5  TS4-iPS clone1 p8  TS4-iPS clone2 p9 |

**Supplementary Table 3** **X chromosome DNA fingerprinting for cell lines used in this study**

| Loci | TS1 | WT-1 | WT-2 | TS-2 | TS-3 | TS-4 |
| --- | --- | --- | --- | --- | --- | --- |
| DXS10103 | 17 | 16/19 | 16/17 | 19 | 16 | 16 |
| DXS8378 | 10 | 10/12 | 10/10 | 11 | 11 | 12 |
| DXS7132 | 14 | 13/14 | 14/15 | 13 | 14 | 14 |
| DXS10134 | 31 | 36/38.3 | 36/38 | 34 | 36 | 36 |
| DXS10074 | 16 | 18/20 | 16/18 | 17 | 16 | 16 |
| DXS10101 | 30 | 28.2 | 28/33 | 32.2 | 31 | 33 |
| DXS10135 | 19 | 16/30 | 20/26 | 19 | 20 | 20 |
| DXS7423 | 15 | 14/15 | 15 | 15 | 15 | 15 |
| DXS10146 | 29 | 26/46.2 | 26/30 | 30 | 28 | 31 |
| DXS10079 | 19 | 18/21 | 20 | 22 | 19 | 22 |
| HPRTB | 13 | 12/13 | 12/14 | 12 | 14 | 14 |
| DXS10148 | 25.1 | 18/23.1 | 28.1 | 24.1 | 23.1 | 28.1 |
| Amelogenin | X | X | X | X | X | X |
