# Supplementary material for: Uniparental disomy of the entire X chromosome in Turner syndrome patient-specific induced pluripotent stem cells
Source: Cell Discov. 2015 Aug 25;1:15022–. doi: 10.1038/celldisc.2015.22 (PMC4860828; doi:10.1038/celldisc.2015.22)
Supplement: Supplementary Figures [file celldisc201522-s2.ppt]

## Slide 1
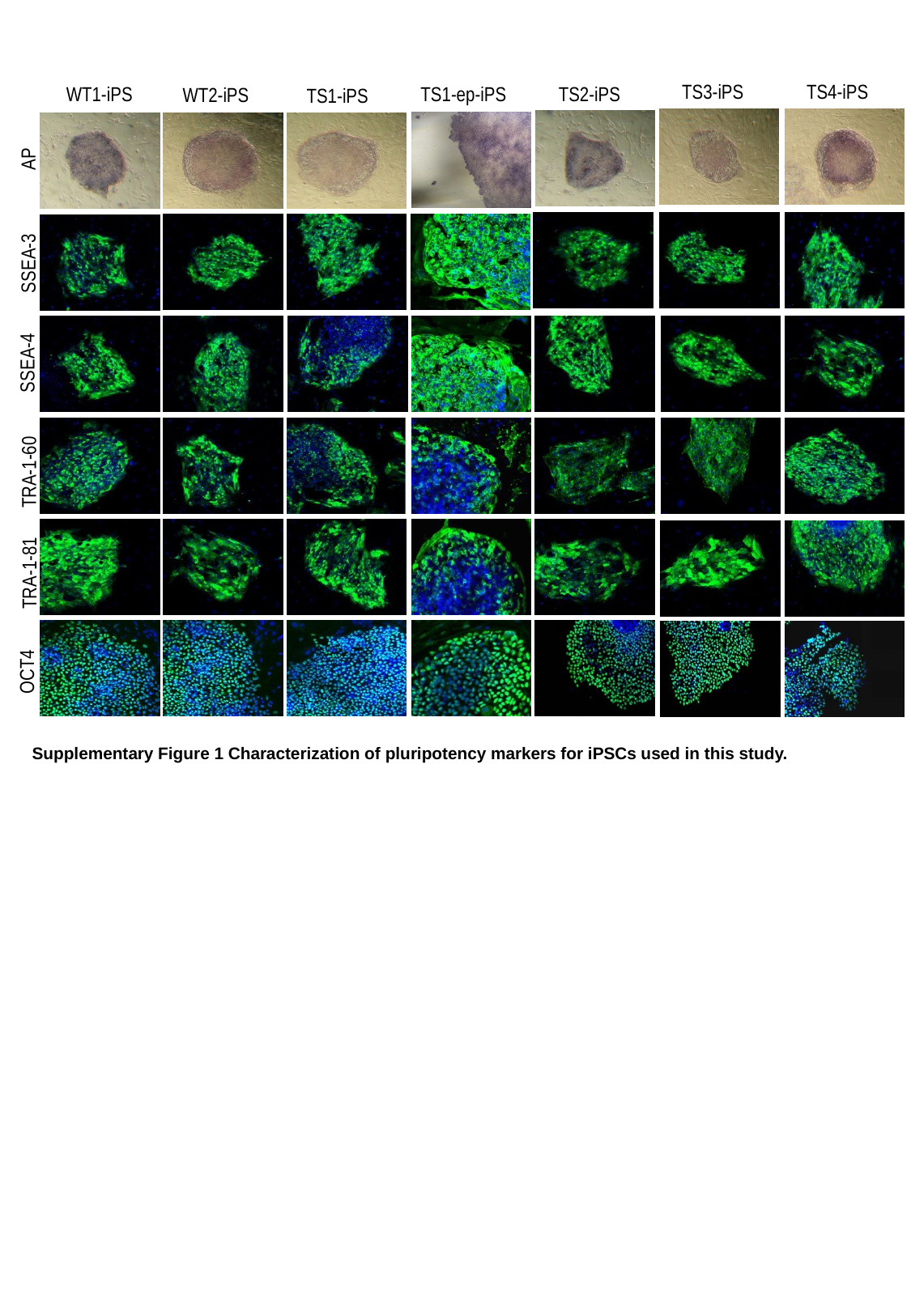

TS3-iPS
TS4-iPS
WT1-iPS
TS1-ep-iPS
TS2-iPS
WT2-iPS
TS1-iPS
AP
SSEA-3
SSEA-4
TRA-1-60
TRA-1-81
OCT4
Supplementary Figure 1 Characterization of pluripotency markers for iPSCs used in this study.

## Slide 2
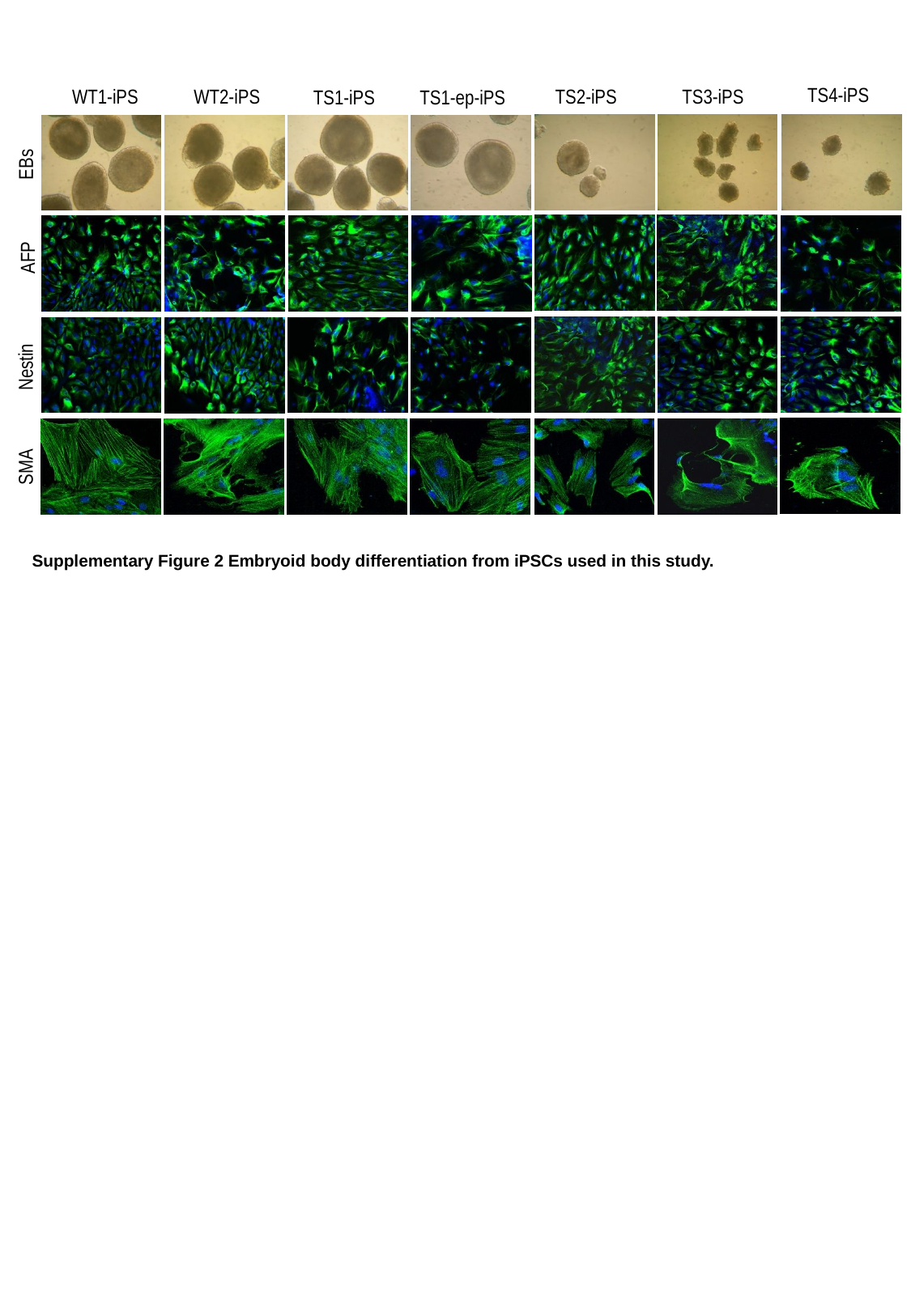

TS4-iPS
WT1-iPS
WT2-iPS
TS2-iPS
TS3-iPS
TS1-iPS
TS1-ep-iPS
EBs
AFP
Nestin
SMA
Supplementary Figure 2 Embryoid body differentiation from iPSCs used in this study.

## Slide 3
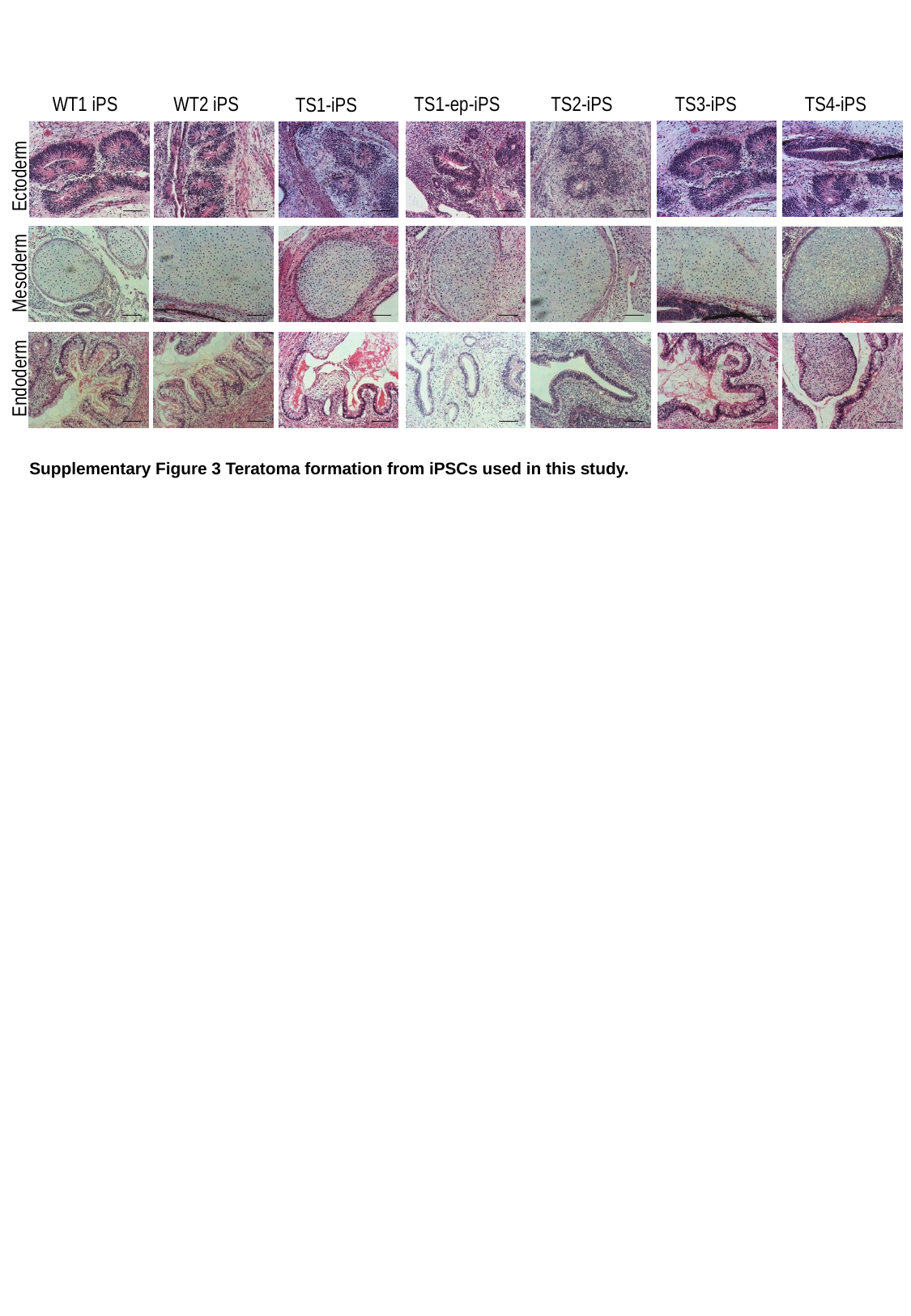

TS1-ep-iPS
TS2-iPS
TS3-iPS
TS4-iPS
WT1 iPS
WT2 iPS
TS1-iPS
Ectoderm
Mesoderm
Endoderm
Supplementary Figure 3 Teratoma formation from iPSCs used in this study.

## Slide 4
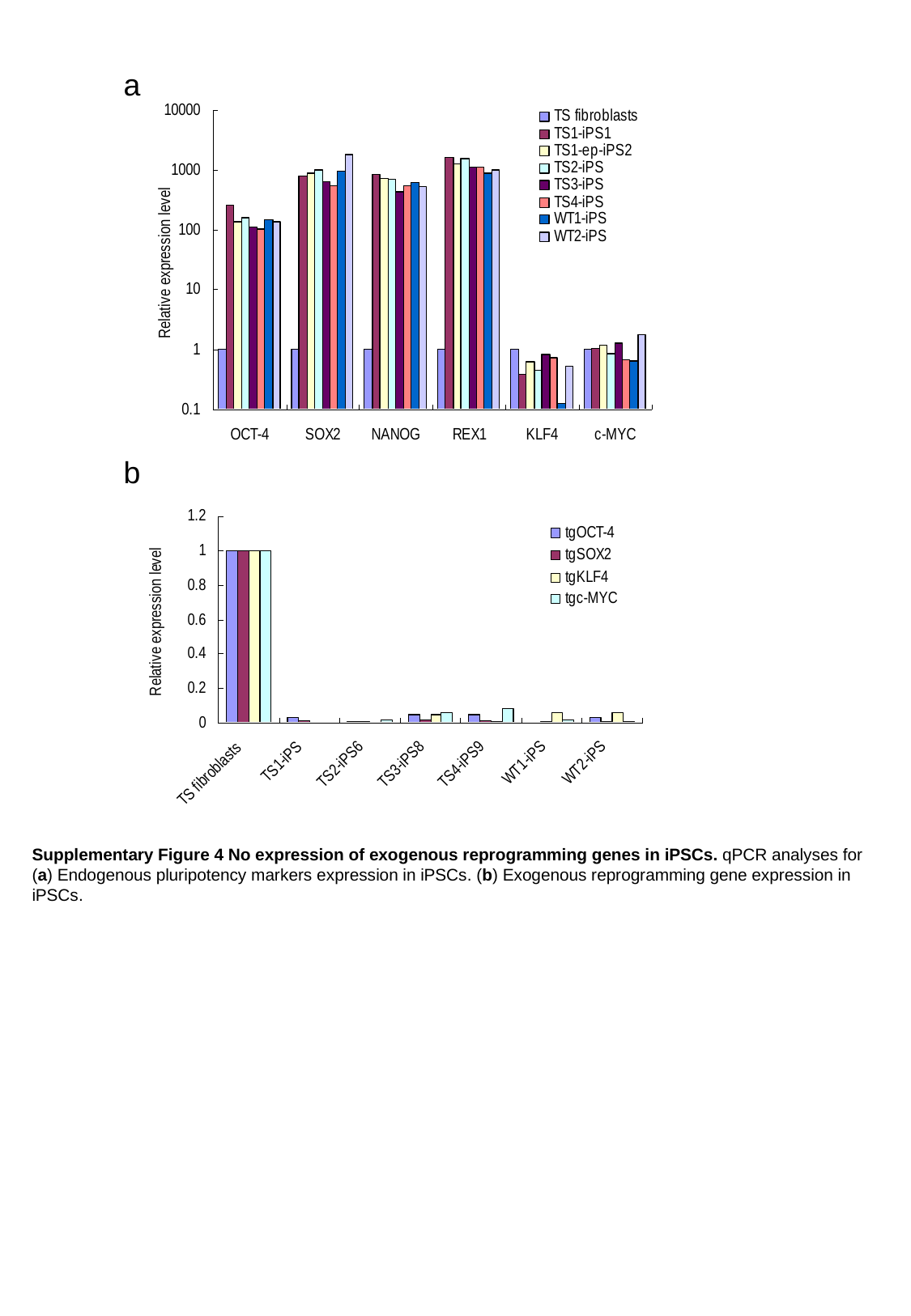

a
b
Supplementary Figure 4 No expression of exogenous reprogramming genes in iPSCs. qPCR analyses for (a) Endogenous pluripotency markers expression in iPSCs. (b) Exogenous reprogramming gene expression in iPSCs.

## Slide 5
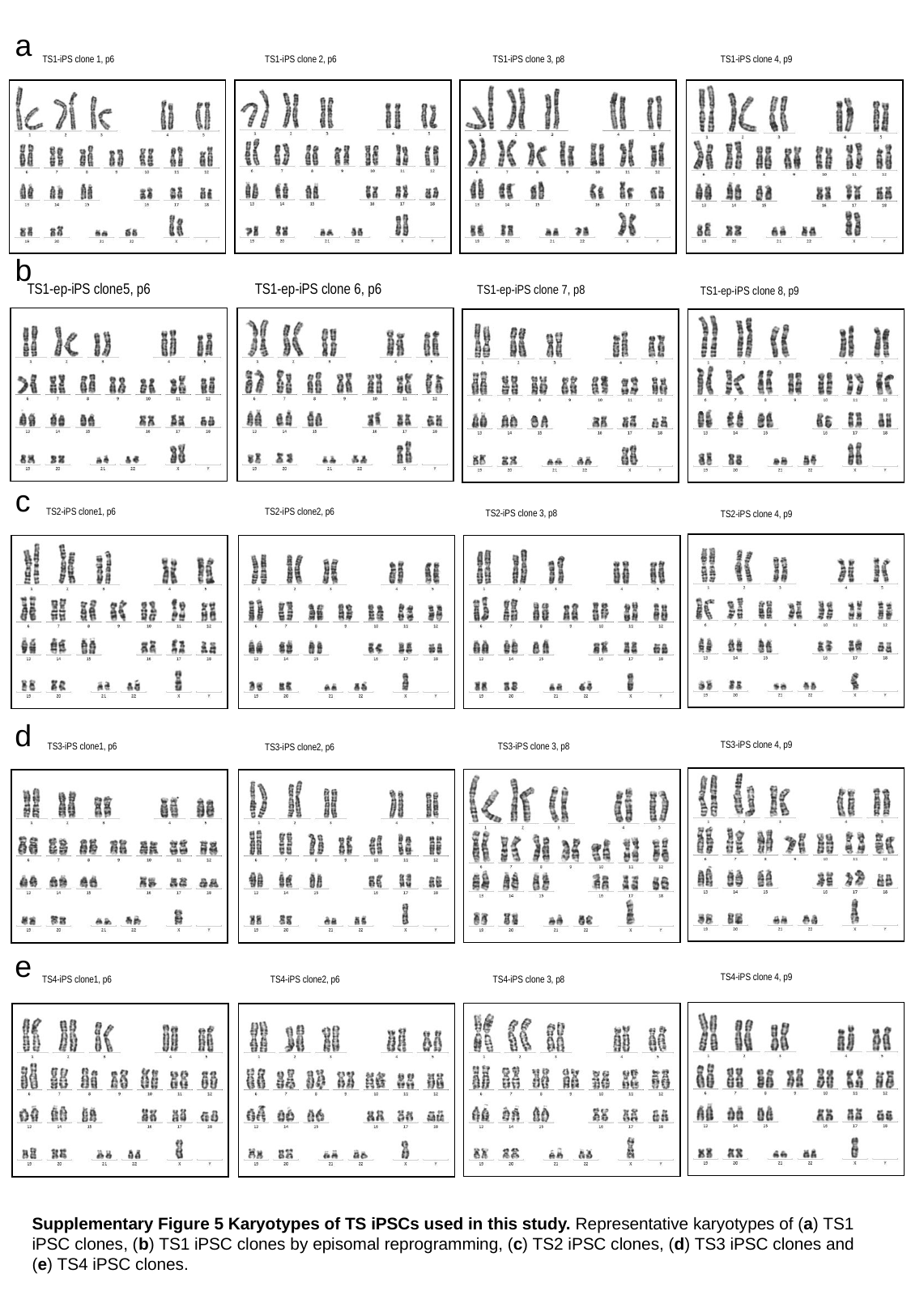

a
TS1-iPS clone 1, p6
TS1-iPS clone 2, p6
TS1-iPS clone 3, p8
TS1-iPS clone 4, p9
b
TS1-ep-iPS clone5, p6
TS1-ep-iPS clone 6, p6
TS1-ep-iPS clone 7, p8
TS1-ep-iPS clone 8, p9
c
TS2-iPS clone1, p6
TS2-iPS clone2, p6
TS2-iPS clone 3, p8
TS2-iPS clone 4, p9
d
TS3-iPS clone 4, p9
TS3-iPS clone1, p6
TS3-iPS clone 3, p8
TS3-iPS clone2, p6
e
TS4-iPS clone 4, p9
TS4-iPS clone 3, p8
TS4-iPS clone1, p6
TS4-iPS clone2, p6
Supplementary Figure 5 Karyotypes of TS iPSCs used in this study. Representative karyotypes of (a) TS1 iPSC clones, (b) TS1 iPSC clones by episomal reprogramming, (c) TS2 iPSC clones, (d) TS3 iPSC clones and (e) TS4 iPSC clones.

## Slide 6
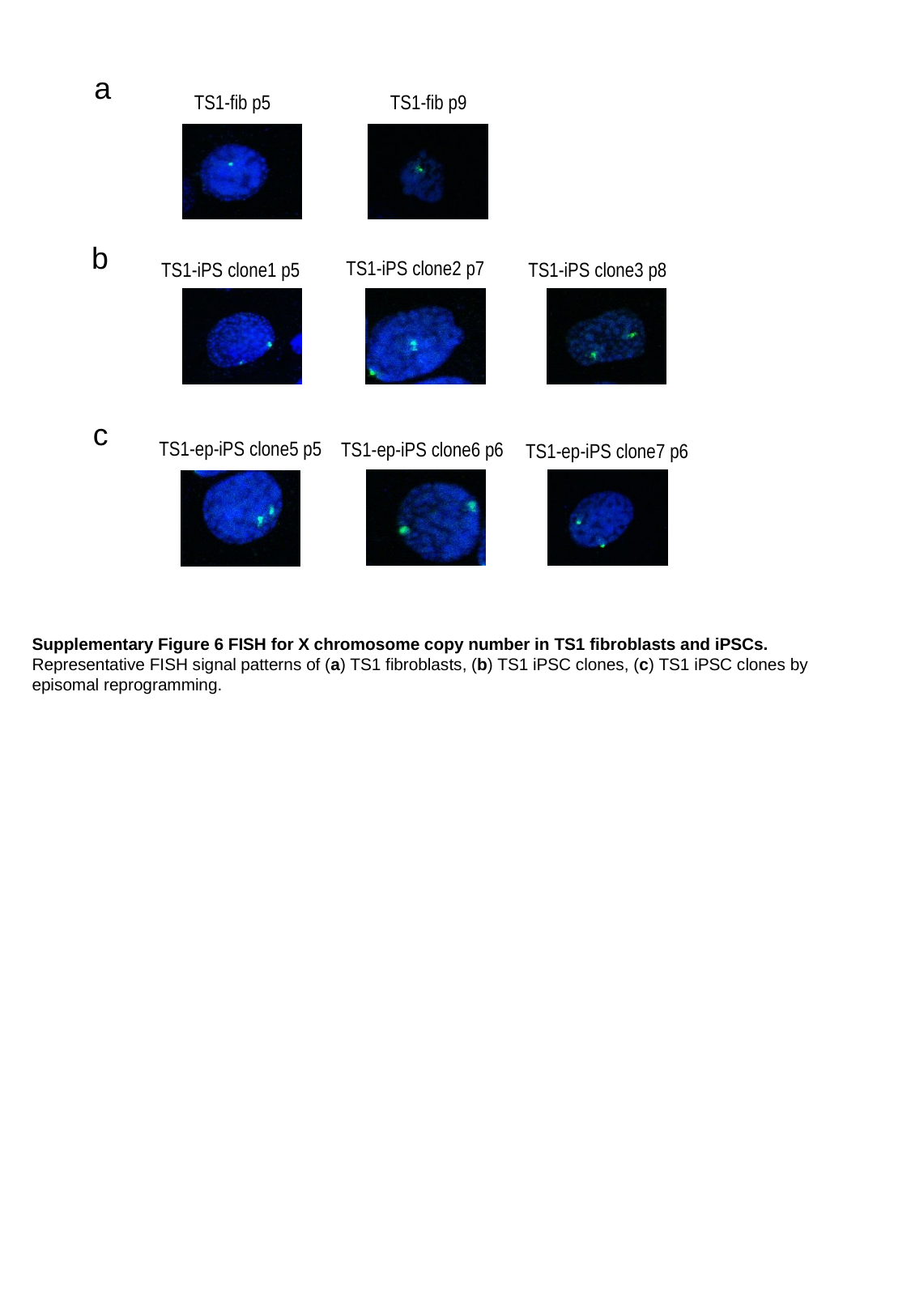

a
TS1-fib p5
TS1-fib p9
b
TS1-iPS clone2 p7
TS1-iPS clone1 p5
TS1-iPS clone3 p8
c
TS1-ep-iPS clone5 p5
TS1-ep-iPS clone6 p6
TS1-ep-iPS clone7 p6
Supplementary Figure 6 FISH for X chromosome copy number in TS1 fibroblasts and iPSCs. Representative FISH signal patterns of (a) TS1 fibroblasts, (b) TS1 iPSC clones, (c) TS1 iPSC clones by episomal reprogramming.

## Slide 7
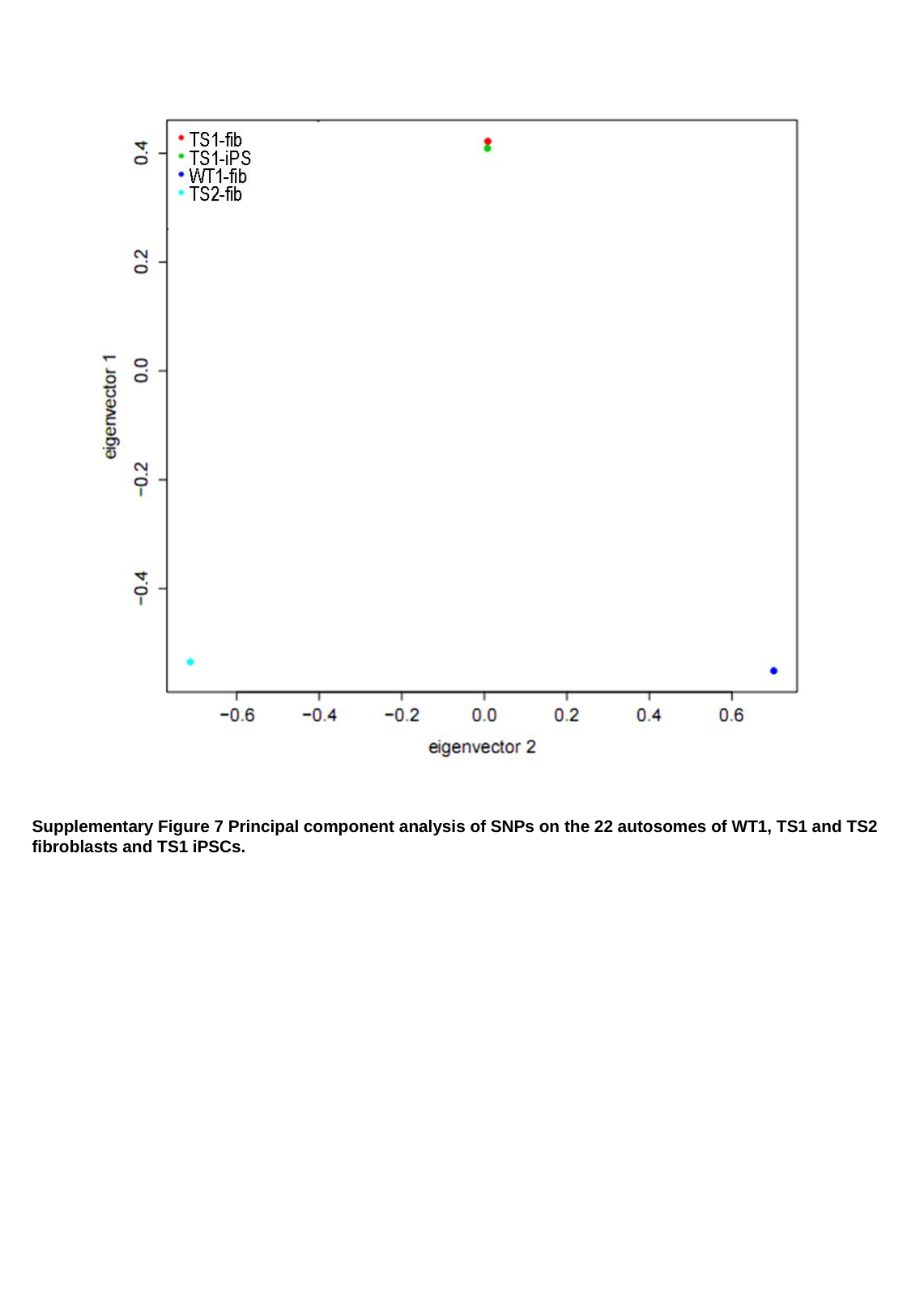

Supplementary Figure 7 Principal component analysis of SNPs on the 22 autosomes of WT1, TS1 and TS2 fibroblasts and TS1 iPSCs.

## Slide 8
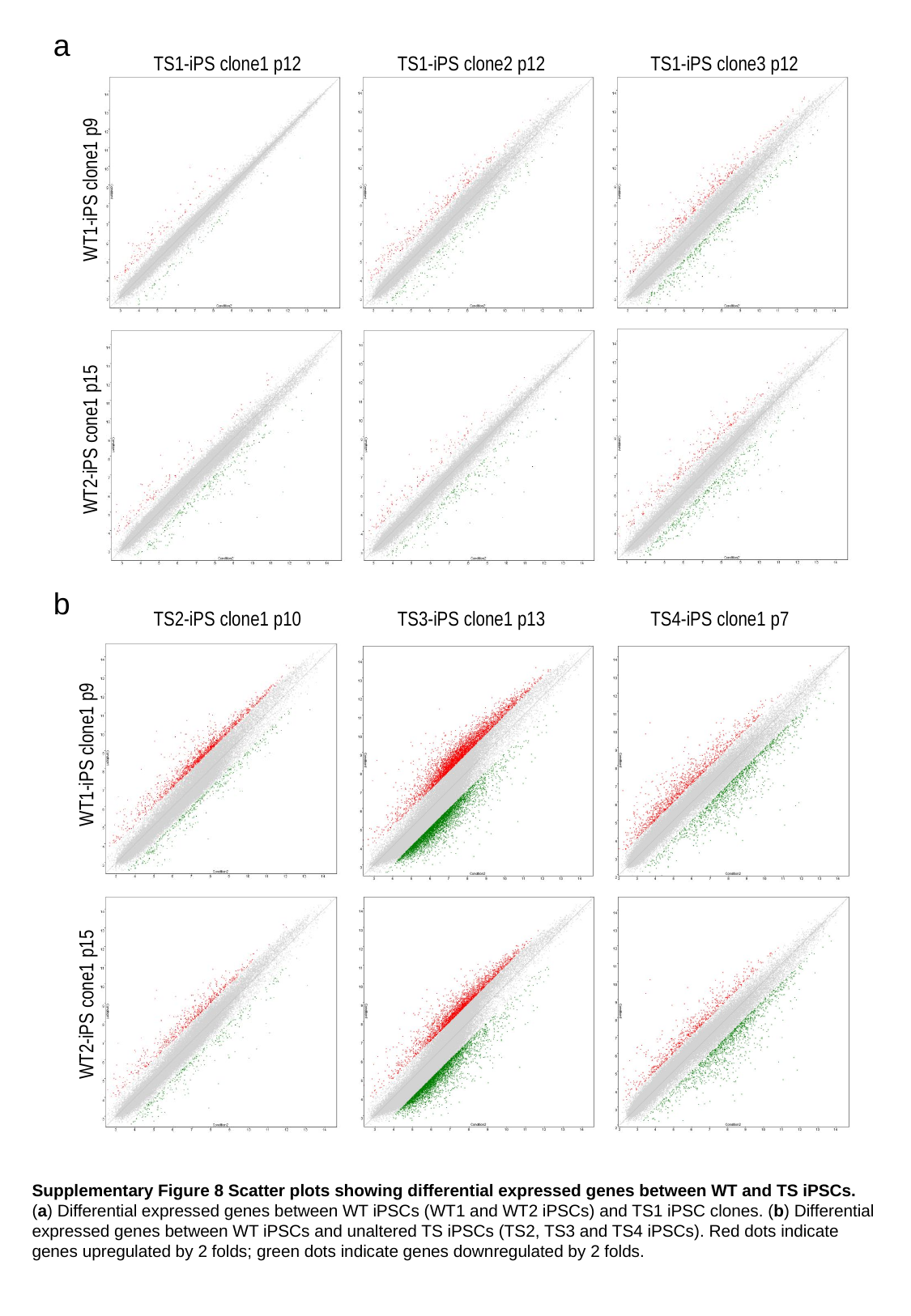

a
TS1-iPS clone2 p12
TS1-iPS clone3 p12
TS1-iPS clone1 p12
WT1-iPS clone1 p9
WT2-iPS cone1 p15
b
TS3-iPS clone1 p13
TS4-iPS clone1 p7
TS2-iPS clone1 p10
WT1-iPS clone1 p9
WT2-iPS cone1 p15
Supplementary Figure 8 Scatter plots showing differential expressed genes between WT and TS iPSCs. (a) Differential expressed genes between WT iPSCs (WT1 and WT2 iPSCs) and TS1 iPSC clones. (b) Differential expressed genes between WT iPSCs and unaltered TS iPSCs (TS2, TS3 and TS4 iPSCs). Red dots indicate genes upregulated by 2 folds; green dots indicate genes downregulated by 2 folds.

## Slide 9
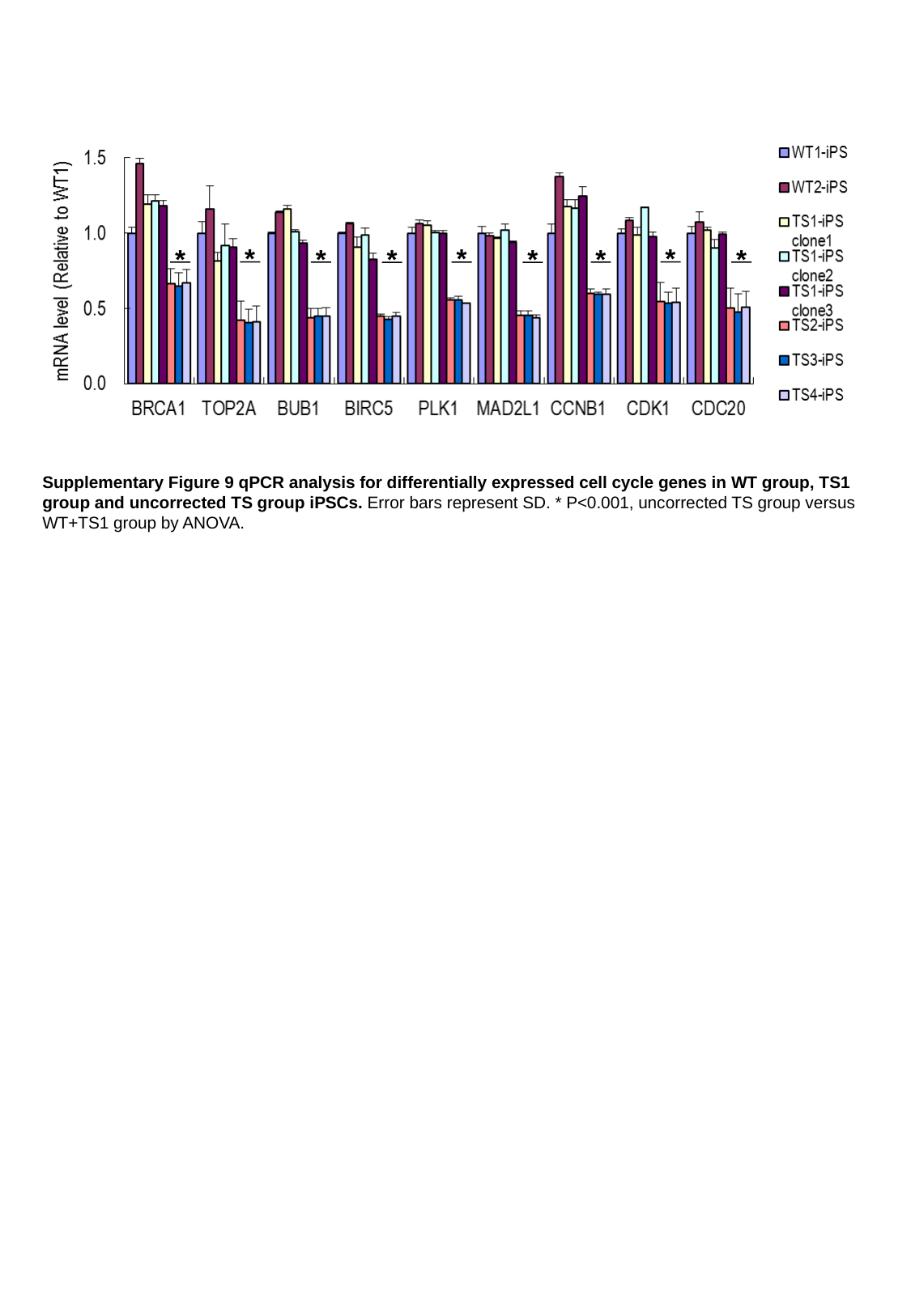

*
*
*
*
*
*
*
*
*
Supplementary Figure 9 qPCR analysis for differentially expressed cell cycle genes in WT group, TS1 group and uncorrected TS group iPSCs. Error bars represent SD. * P<0.001, uncorrected TS group versus WT+TS1 group by ANOVA.
